# Mycobacterium marinum Infection Leading to HIV Diagnosis: A Case Report

**DOI:** 10.7759/cureus.73016

**Published:** 2024-11-04

**Authors:** Ayaka Yasuda, Natsuko Saito-Sasaki, Risa Nakane, Haruna Yoshioka, Yu Sawada

**Affiliations:** 1 Dermatology, University of Occupational and Environmental Health, Kitakyushu, JPN; 2 Dermatology, Japan Community Healthcare Organization Kyushu Hospital, Kitakyushu, JPN

**Keywords:** case report, hiv, mycobacterium marinum, nontuberculous mycobacteriosis, skin

## Abstract

Non-tuberculous mycobacteriosis (NTM) is a rare infection that can manifest as pulmonary, disseminated, or cutaneous disease. Cutaneous NTM infections are often associated with immunosuppressive conditions, such as HIV infection or exposure to contaminated water, but they can also occur in healthy individuals, complicating the diagnosis. We report the case of a 54-year-old male with a gradually enlarging skin ulcer on his left upper limb. Initial treatment for sporotrichosis failed, and further testing confirmed *Mycobacterium marinum* infection. The patient had no prior medical history, but due to the persistent nature of the symptoms, HIV testing was performed, revealing advanced HIV infection with a CD4 count of 34 cells/µl. Following appropriate antimicrobial treatment, the ulcer healed, and antiretroviral therapy (ART) was initiated, resulting in a significant improvement in immune function. This case highlights the importance of considering underlying immunosuppressive conditions, such as HIV, when diagnosing persistent cutaneous NTM infections. Early detection and prompt treatment of both *M. marinum* and HIV were critical in preventing the progression to disseminated disease, which is often fatal in immunocompromised patients.

## Introduction

Non-tuberculous mycobacteriosis (NTM) is a rare infection that can present in different forms, including pulmonary, disseminated, or cutaneous disease [1,2]. Cutaneous NTM infections are commonly associated with immunosuppressive conditions, such as HIV infection or environmental exposure to contaminated water or soil. Other risk factors include using immunosuppressive medications, other immunosuppressive factors such as diabetes, and advanced age [3,4]. However, it can also occur in patients who do not exhibit obvious risk factors, making diagnosis less straightforward [5].

*Mycobacterium marinum* accounts for most NTM skin infections [6]. However, cutaneous NTM infections can arise from various species and are often associated with immunosuppression [7]. In this report, we present the case of a patient who was initially suspected of having an *M. marinum* infection. Although the patient was considered to be in good health, further investigations led to the diagnosis of advanced HIV infection, as indicated by a CD4 cell count of 34 cells/μL and a high viral load of 370,000 copies/mL.

## Case presentation

**Figure 1:**
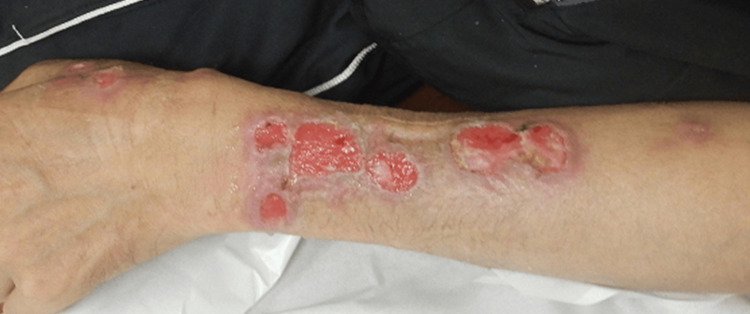
Clinical presentation A well-demarcated skin ulcer surrounded by erythematous tissue, with multiple nodular and granulomatous lesions, extending from the patient's left hand to the forearm.

A 54-year-old male noticed a gradually enlarging skin ulcer on his left upper limb. Initially, he visited a local clinic where sporotrichosis was suspected based on the linear pattern of the ulcer, and he was prescribed oral itraconazole for one month. However, the condition did not improve; therefore, he was referred to our hospital to evaluate his skin eruption. Upon examination, he presented with a well-demarcated skin ulcer surrounded by erythematous tissue with multiple roundish ulcers resembling a nodular granulomatous pattern (Figure 1).

The patient had no significant past medical history or medication history. Notably, he had been keeping tropical fish at home, which raised suspicion of *M. marinum* infection. A culture for acid-fast bacilli was performed, confirming the presence of *M. marinum*.

Laboratory results showed the following: His white blood cell count was 4000/ml, with 68.7% neutrophils, 4.6% eosinophils, 0.8% basophils, 17.5% lymphocytes, and 8.4% monocytes. Hemoglobin was 13.1 g/dl, and platelets were 248,000/μl. The β-D-glucan level was slightly elevated at 14.1 pg/ml. Cytomegalovirus antigenemia was positive, but T-SPOT and *Mycobacterium avium* complex (MAC) (*M. avium* and *M. intracellulare*) antibodies were negative.

**Figure 2:**
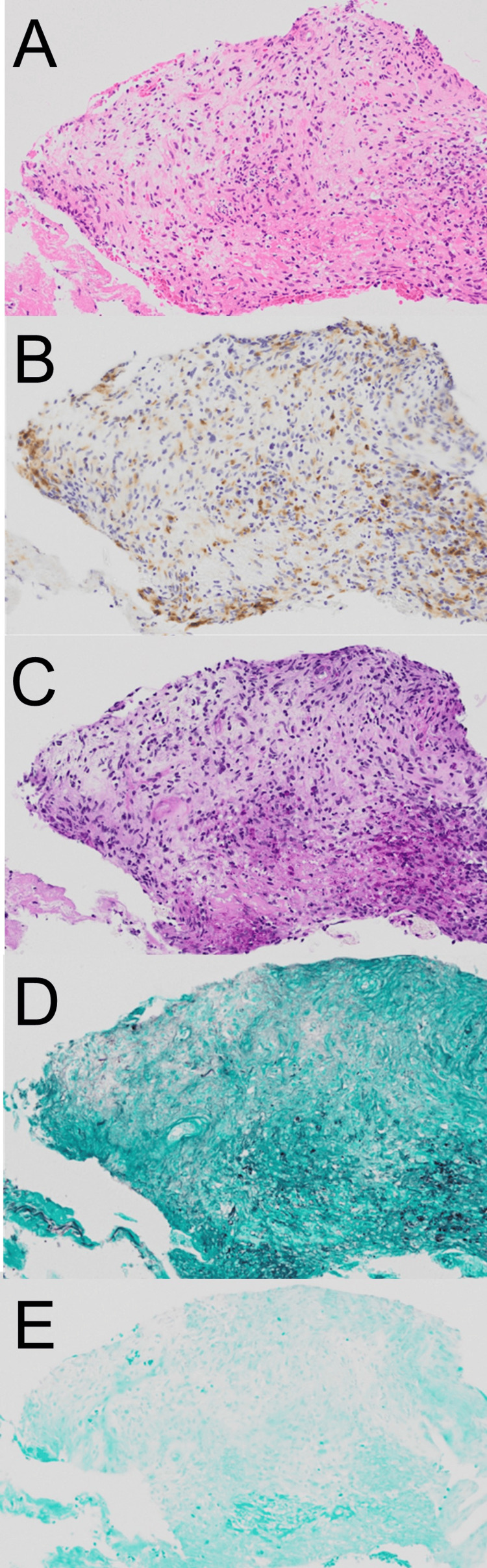
Histological examination (A) The specimen shows inflammatory granulation tissue with scattered or partially aggregated histiocytes, necrotic debris, and inflammatory exudate associated with subcorneal abscess formation in the acanthotic epidermis. (B) Immunostaining for CD68, highlighting macrophages consistent with a granulomatous inflammatory response. (C) PAS (periodic acid-Schiff) staining shows no fungal organisms. (D) Grocott's methenamine silver staining reveals no evidence of fungal elements. (E) Ziehl-Neelsen staining for acid-fast bacilli shows no identifiable mycobacteria. No evidence of malignancy is present in the specimen.

The wound culture did not show bacterial growth, and PCR tests for *M. avium* and *M. intracellulare* were negative. However, MALDI-TOF MS identified *M. marinum* as the causative organism. At his first examination at our hospital, a linear chain of ulcers was observed extending from the back of his left hand to his forearm, each ulcer roughly the size of a thumb. Histopathological examination showed histiocytes forming granulomas surrounding the ulcer structure. No fungi were detected with PAS or Grocott staining, and Ziehl-Neelsen staining for acid-fast bacilli was negative (Figure 2A-2E).

Given the persistence of his symptoms and the lack of improvement, further investigations were performed, including HIV testing. The results confirmed HIV-1 infection with a viral load of 370,000 copies/ml, a CD4 count of 34 cells/μl, and a CD4/CD8 ratio of 0.1. This late diagnosis of advanced HIV infection helped explain the severity of his cutaneous NTM infection.

Based on the drug sensitivity test results, the patient was prescribed levofloxacin, and the ulcer healed. After four weeks of treatment, the ulcer epithelialized, and the patient stopped taking the medication. Half a month later, cytomegalovirus (CMV) enteritis was diagnosed through a lower gastrointestinal endoscopy. Two and a half months after his first visit to our hospital, he began treatment for HIV with a combination of bictegravir, tenofovir alafenamide, and emtricitabine. The HIV-1 count began to decrease, and the CD4 lymphocyte count increased. The patient was also diagnosed with *M. marinum* infection associated with AIDS.

## Discussion

*M. marinum* infections account for the largest proportion of nontuberculous mycobacterial skin infections. They are most common in persons who handle fish or keep tropical fish. The skin lesions initially appear as red papules or pustules, and as they enlarge, they become ulcerated or form subcutaneous abscesses, nodules, or masses in the center. A study examining 27 cases of *M. marimum* infection found that about 44% involved exposure to fish tanks or fishing activities [8]. Surgical excision and thermotherapy are performed in addition to administering antibacterial drugs.

*M. marinum* infections account for the largest proportion of nontuberculous mycobacterial skin infections. They are most common in persons who handle fish or keep tropical fish. The skin lesions initially appear as red papules or pustules, and as they enlarge, they become ulcerated or form subcutaneous abscesses, nodules, or masses in the center. A study examining 27 cases of *M. marimum* infection found that about 44% involved exposure to fish tanks or fishing activities [8]. Surgical excision and thermotherapy are performed in addition to administering antibacterial drugs.

In acquired immunodeficiency syndrome (AIDS)-related diseases, nontuberculous mycobacterial infections often present as disseminated, affecting areas beyond the lungs, skin, neck, or hilar lymph nodes. HIV patients become more vulnerable to opportunistic infections, such as disseminated forms of *M. avium* and *M. intracellulare*, as CD4 lymphocyte counts drop below 200/mm³ [9]. *M. marinum* frequently develops in the extremities, such as the fingers, which are prone to trauma, and lower temperatures in these regions are considered to limit the potential for systemic dissemination [10]. Disseminated *M. marinum* infections can extend beyond localized lesions, affecting multiple organs and tissues, particularly in individuals with compromised immune function, such as HIV patients or transplant recipients.

The literature documents two cases of disseminated *M. marinum* infection in immunosuppressed patients. Both patients receiving infliximab for Crohn's disease developed widespread *M. marinum* infections after exposure to water [11,12]. These cases highlight the risk of systemic dissemination in immunosuppressed individuals, particularly those undergoing biologic therapy.

Furthermore, *M. marinum* infection was diagnosed after HIV infection had already been identified. A renal transplant patient with long-term HIV developed a disseminated *M. marinum* infection despite being on antiretroviral therapy (ART) [13]. Thus, the patient's HIV status was already known before the diagnosis of the *M. marinum* infection. Currently, there are no widely documented cases where the diagnosis of *M. marinum* has directly led to the discovery of an underlying HIV infection. However, as in our case, considering the possibility of HIV co-infection may help prevent the progression to disseminated disease. Early recognition and intervention can be crucial in managing *M. marinum* infections, particularly in immunocompromised patients, and they may reduce the risk of systemic spread.

In this case, early suspicion of an *M. marinum* infection followed by prompt diagnostic testing allowed for timely intervention. Furthermore, the rapid diagnosis of HIV infection enabled treatment before the infection could systemically disseminate. Early detection of both *M. marinum* and HIV was crucial in preventing the progression to disseminated disease, allowing successful treatment.

## Conclusions

We encountered a case where HIV infection was diagnosed following suspicion of *M. marinum* infection. *M. marinum* typically causes localized skin infections but can spread and become life-threatening in immunocompromised individuals. Early diagnosis of HIV allowed for prompt treatment, preventing the infection from progressing to a more dangerous, disseminated form. This case highlights the importance of timely intervention in HIV-positive patients, where immunosuppression can worsen otherwise mild infections. It also underscores the need for immune evaluations, including HIV testing, in patients with suspected atypical mycobacterial infections. While *M. marinum* infection is a serious concern, detecting and treating the underlying HIV infection is equally, if not more, important, especially since HIV compromises the immune system, making the patient more susceptible to various opportunistic infections. In this context, early detection of HIV is crucial not only for managing the *M. marinum* infection but also for initiating ART to control the HIV infection. The goal is to strengthen the immune system and prevent further complications. While *M. marinum* can cause localized or disseminated infections, untreated HIV has far-reaching consequences, including a heightened risk of other opportunistic infections, and without treatment, it could lead to AIDS and life-threatening complications. It might be useful to clarify in the conclusion that recognizing and treating HIV early has dual importance: It enables better control of infections like *M. marinum*. It is vital for long-term immune function and overall health by preventing the progression of AIDS.
